# Early antithrombotic post-discharge therapy using prophylactic DOAC or dipyridamole improves long-term survival and cardiovascular outcomes in hospitalized COVID-19 survivors

**DOI:** 10.3389/fcvm.2022.916156

**Published:** 2022-07-29

**Authors:** Lukas J. Motloch, Peter Jirak, Moritz Mirna, Lukas Fiedler, Paruir A. Davtyan, Irina A. Lakman, Diana F. Gareeva, Anton V. Tyurin, Ruslan M. Gumerov, Simon T. Matskeplishvili, Valentin N. Pavlov, Benzhi Cai, Kristen Kopp, Albert Topf, Uta C. Hoppe, Rudin Pistulli, Naufal S. Zagidullin

**Affiliations:** ^1^Clinic II for Internal Medicine, University Hospital Salzburg, Paracelsus Medical University, Salzburg, Austria; ^2^Department of Internal Medicine, Cardiology, Nephrology and Intensive Care Medicine, Hospital Wiener Neustadt, Wiener Neustadt, Austria; ^3^Department of Internal Medicine I, Bashkir State Medical University, Ufa, Russia; ^4^Scientific Laboratory for the Study of Socio-Economic Problems of the Regions Bashkir State University, Ufa, Russia; ^5^Department of Internal Diseases II, Bashkir State Medical University, Ufa, Russia; ^6^Lomonosov Moscow State University Medical Center, Moscow, Russia; ^7^Department of Urology, Bashkir State Medical University, Ufa, Russia; ^8^Department of Pharmacy at The Second Affiliated Hospital, and Department of Pharmacology (The Key Laboratory of Cardiovascular Medicine Research, Ministry of Education) at College of Pharmacy, Harbin Medical University, Harbin, China; ^9^Department of Cardiology I, Coronary and Peripheral Vascular Disease, Heart Failure, University Hospital Munster, Munster, Germany

**Keywords:** COVID-19, long COVID-19, direct anticoagulation, dipyridamole, cardiovascular disease in COVID-19

## Abstract

**Introduction:**

Cardiovascular events are common in COVID-19. While the use of anticoagulation during hospitalization has been established in current guidelines, recommendations regarding antithrombotic therapy in the post-discharge period are conflicting.

**Methods:**

To investigate this issue, we conducted a retrospective follow-up (393 ± 87 days) of 1,746 consecutive patients, hospitalized with and surviving COVID-19 pneumonia at a single tertiary medical center between April and December 2020. Survivors received either 30-day post-discharge antithrombotic treatment regime using prophylactic direct oral anticoagulation (DOAC; *n* = 1,002) or dipyridamole (*n* = 304), or, no post-discharge antithrombotic treatment (Ctrl; *n* = 440). All-cause mortality, as well as cardiovascular mortality (CVM) and further cardiovascular outcomes (CVO) resulting in hospitalization due to pulmonary embolism (PE), myocardial infarction (MI) and stroke were investigated during the follow-up period.

**Results:**

While no major bleeding events occured during follow-up in the treatment groups, Ctrl showed a high but evenly distributed rate all-cause mortality. All-cause mortality (CVM) was attenuated by prophylactic DOAC (0.6%, *P* < 0.001) and dipyridamole (0.7%, *P* < 0.001). This effect was also evident for both therapies after propensity score analyses using weighted binary logistic regression [DOAC: *B* = −3.33 (0.60), *P* < 0.001 and dipyridamole: *B* = −3.04 (0.76), *P* < 0.001]. While both treatment groups displayed a reduced rate of CVM [DOAC: *B* = −2.69 (0.74), *P* < 0.001 and dipyridamole: *B* = −17.95 (0.37), *P* < 0.001], the effect in the DOAC group was driven by reduction of both PE [*B*−3.12 (1.42), *P* = 0.012] and stroke [*B* = −3.08 (1.23), *P* = 0.028]. Dipyridamole significantly reduced rates of PE alone [*B* = −17.05 (1.01), *P* < 0.001].

**Conclusion:**

Late cardiovascular events and all-cause mortality were high in the year following hospitalization for COVID-19. Application of prophylactic DOAC or dipyridamole in the early post-discharge period improved mid- and long-term CVO and all-cause mortality in COVID-19 survivors.

## Introduction

Cardiovascular events including thromboembolisms due to coagulopathy represent frequent and serious complications in COVID-19 patients. Accordingly, high rates of stroke, pulmonary embolism and venous thromboembolisms have been reported in the context of COVID-19 disease. These events seem primarily driven by the profound inflammatory response, along with endothelial inflammation and dysfunction (1–3) which cause an increase in platelet adhesion and aggregation, thus promoting procoagulatory effects and thromboinflammatory processes (3, 4). Additionally, platelet activation itself further triggers the release of proinflammatory cytokines. As a consequence, elevated levels of fibrinogen and D-dimer have been reported as frequent finding of prognostic relevance in COVID-19 patients. Furthermore, occlusive thrombotic micro-angiopathy has been observed (5). While prothrombotic effects in acute COVID-19 disease seem evident, there is conflicting data in this context, as well as a lack of long-term follow-up evaluating the risk of cardiovascular events and death in the post-discharge period (6, 7).

In hospitalized patients, no beneficial effects of therapeutic anticoagulation was observed in critically ill COVID-19 (8, 9), while non-critically ill COVID-19 patients seem to benefit from this therapeutic approach (10–12). Since a higher inflammatory burden is present in critically ill patients, COVID-19-related vascular inflammation was discussed as a potential explanation for these controversial findings (13–16). Early studies investigating the use of antiplatelet agents in acute COVID-19 also showed promising results (17). However, these findings could not be confirmed in large, randomized trials (18, 19). On the other hand, smaller trials indicated a potentially beneficial effect of dipyridamole (20, 21). In addition, although COVID-19 also affects long-term cardiovascular outcomes (22), present antithrombotic guidelines for extended post-discharge thromboprophylaxis are conflicting, recommending either no routine thromboprophylaxis or an individualized approach (23, 24).

In mid-2020, dipyridamole or prophylactic direct anticoagulation (DOAC) were routinely prescribed in the early post-discharge period (30-days post-discharge) in several medical centers based on experts' recommendations. This approach was subsequently adopted in a nationwide class C guideline recommendation for prophylactic DOAC in September 2020 (25). The use of anticoagulants in the post-discharge regime following COVID-19 hospitalization seems to be supported by data from a US registry in the 90 day follow-up of post-discharge COVID-19 patients (7) as well as by recent results from Brazil indicating prophylactic rivaroxaban improves short-term (35 days) outcomes in high-risk patients (26). Nevertheless, to the best of our knowledge, the efficacy and safety of the described strategy have not been systematically or adequately evaluated, despite its routine use in clinical practice. Furthermore, longer follow-up data on cardiovascular outcomes in hospitalized COVID-19 survivors are also lacking. To investigate this issue, we assessed the incidence of all-cause death as well as cardiovascular mortality and hospitalizations for relevant cardiovascular outcomes including pulmonary embolism, stroke and myocardial infarction of 1,746 hospitalized COVID-19 survivors receiving post-discharge thromboprophylaxis using either prophylactic DOAC or dipyridamole or no thromboprophylaxis during follow-up of 393 ± 87 days. We hypothesized, that the applied thromboprophylactic post-discharge strategy would affect incidence of cardiovascular events and thus potentially all-cause mortality rates.

## Methods

The study was performed in accordance with the standards of good clinical practice and the principles of the Declaration of Helsinki, receiving approval by the ethics commission of the Bashkir State Medical University (N5, 2020).

For this single-center, retrospective study, 2,294 COVID-19 survivors were consecutively screened at discharge following hospitalization for COVID-19 disease at a tertiary medical center (Bashkir State Medical University Hospital, Bashkir State, Russian Federation) between April 2020 and December 2020 for moderate COVID-19 associated pneumonia, defined according to current WHO recommendations (27).

**Figure 1:**
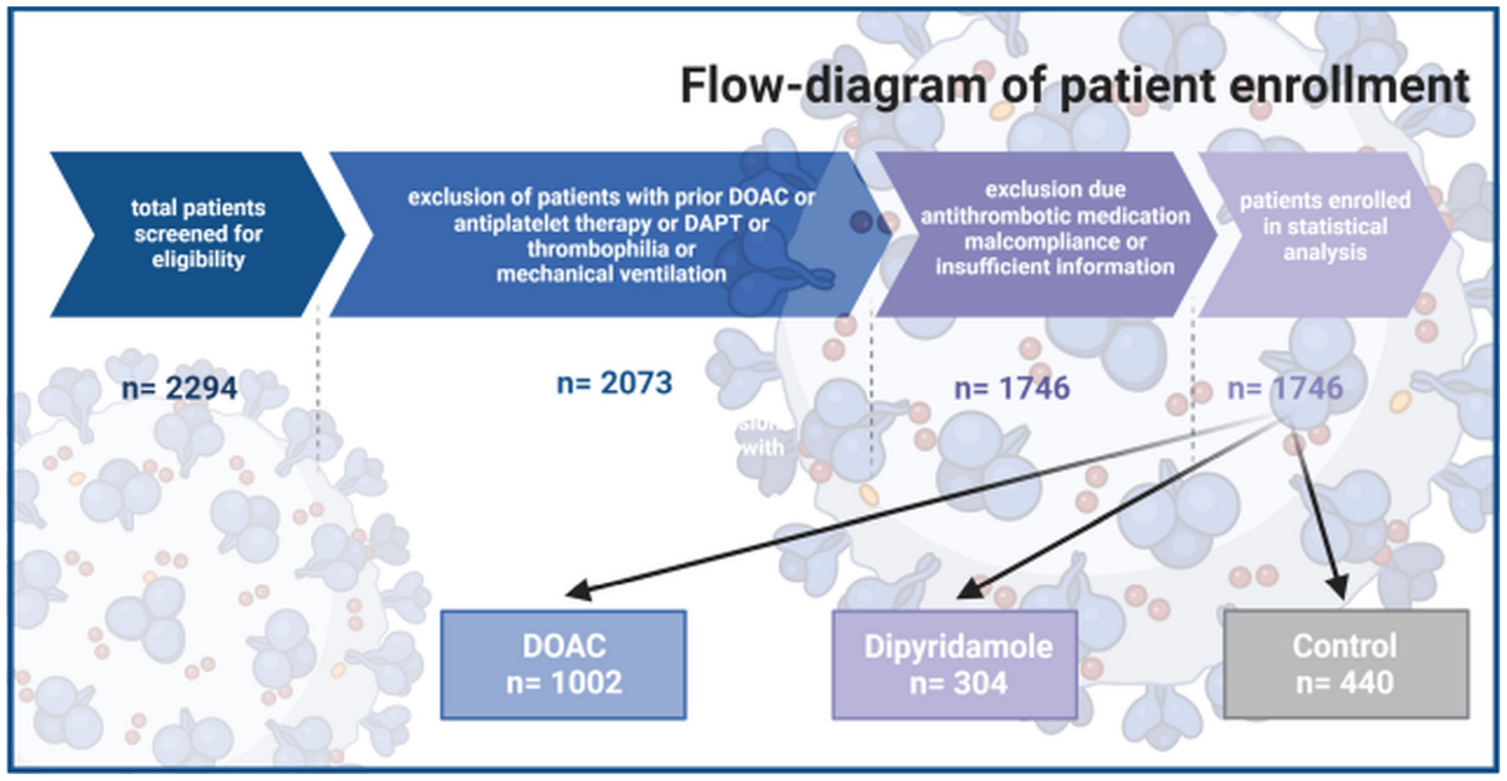
Flow chart of patients' inclusion

All included patients were 18 years or older and suffered from moderate COVID-19-related pneumonia requiring hospitalization. Exclusion criteria were defined as: requirement for therapeutic anticoagulation using Vitamin K antagonists or therapeutic DOAC therapy before or/and after enrollment, history of relevant thrombotic disorders requiring anticoagulation therapy. Furthermore, with respect to potential bleeding complication, according to our hospital standard of clinical care procedures, patients with requirement for combination therapy of DOAC and/or dipyridamole and/or any other additional antiplatelet therapies including acetylsalicylic acid, ticagrelor, prasugrel or clopidogrel were not considered for the investigated post-discharge antithrombotic regimes. Consequently, to avoid any bias, which might be associated with the described patients' selection, patients in Ctrl treated with antiplatelet medications including acetylsalicylic acid, ticagrelor, prasugrel or clopidogrel were also excluded from further analyses. In addition, to account for disease severity and associated potential thrombotic risk, patients requiring mechanical ventilation during their hospitalization were also excluded from further analyses (Figure 1).

Based on the primary inclusion and exclusion criteria, 2,073 qualifying COVID-19 patients were further stratified based upon the recommended anticoagulation post-discharge regime: anticoagulation using dipyridamole 75 mg TID or prophylactic DOAC (DOAC) for 30-days post hospital discharge (rivaroxaban 10 mg QD, dabigatran 110 mg BID, or apixaban 2.5 mg BID) or a no anticoagulation therapy group as the control group (Figure 1). Choice of the antithrombotic post-discharge therapy (Ctrl. or specific DOAC or dipyridamole) was based on the decision of the attending physician and implemented hospital-specific standard of care procedures. In all eligible patients, patient hospital data including demographics, medical history, laboratory examinations, comorbidities, complications, specific treatment measures, and outcomes were collected and analyzed. During follow-up, patients' outcome and survival were evaluated until October 1, 2021. The investigated outcomes were compromised of all-cause mortality and the need for hospitalization due to cardiovascular events including pulmonary embolism, myocardial infarction and stroke. We further analyzed the incidence of cardiovascular mortality defined as in hospital death due to cardiovascular causes or out of hospital death meeting the criteria of sudden cardiac death (28). In addition, patients were evaluated for relevant bleeding events requiring hospitalization. Major and non-major bleeding, were defined according to the International Society on Thrombosis and Haemostasis (ISTH) criteria (29). Follow-up was conducted with the help of the remote data capture system “ProMed” (Program for Medical Cases Monitoring). The program enables distant online monitoring of all hospitalization discharge notes of all regional hospital institutions as well as all death certificates.

At the time point of data collection (after October 1st 2021), all patients with confirmed recommendation for post-discharge anticoagulation were further contacted by phone. A standardized telephone interview was performed to verify the applied antithrombotic substance use and to confirm compliance to the DOAC or dipyridamole regime in the post-discharge setting (DOAC. including rivaroxaban, dabigatran and apixaban or dipyridamole). If a patient was deceased by the time of scheduled contact, a standardized telephone interview was performed with a close relative. Patients were excluded from further analyses, if the recommended anticoagulation regime was not taken by the patient or if collection of sufficient information about the therapy regime was not possible (*n* = 327). Follow-up outcomes in the remaining 1,002 patients with confirmed prophylactic Direct Oral Anticoagualtion (DOAC) intake and 304 patients with confirmed dipyridamole therapy intake was propensity-matched to the control group, in whom no anticoagulation regime was prescribed at hospital discharge (Figure 1).

### Statistical analyses

Statistical analyses were conducted using R [version 4.0.2., R Core Team (2013), R Foundation for Statistical Computing, Vienna, Austria; http://www.R-project.org/] and the packages “Rcmdr,” “ggplot2,” “pastecs,” “Hmisc,” “ggm,” “polycor,” “QuantPsyc,” “glmnet,” “twang,” “survey,” “stddidff,” “survival” and “survminer,” as well as, SPSS (Version 23.0, IBM, Armonk, New York, USA). Distribution of continuous data was assessed visually and using the Kolmogorov-Smirnov-test, kurtosis and skew were assessed visually. Since data were not normally distributed, median ± interquartile-range (IQR) are depicted. Medians were compared by Kruskal-Wallis test, whereas categorical data was analyzed using Fisher's exact test. Survival probability is depicted using the Kaplan-Meier method, Cox proportional hazards analysis was performed to assess the association of applied therapies with mortality. To account for imbalances in baseline covariates with possible influence on outcome, standardized differences between the three groups were calculated. Covariates with statistically significant differences or standardized differences >0.25 between the groups (see Table 1; arterial hypertension, age, C-reactive protein, in-hospital treatment with corticosteroids, in-hospital treatment with anticoagulation, in-hospital treatment with IL-6 antagonists) were then included in propensity score weighting of the groups by Generalized Boosted Models (GBM) using the Average Treatment Effect on Treated (ATT) estimate (30). Prior to GBM, continuous data were transformed to z-scores to assure standardization and overlap concerns were checked by density plots of continuous data, as well as cross tabulations of nominal data. After balancing, weighted logistic regression analysis was performed for the predefined endpoints of the study using the “survey” package of R. A *p*-value of <0.05 was considered statistically significant.

**Table 1:** Baseline characteristics of enrolled patients.

	**DOAC (*****n*** = **1,002)**		**Dipyridamole (*****n*** = **304)**		**Control (*****n*** = **440)**			
	**%**	***n***	**%**	***n***	**%**	***n***	***P*** **-value**	**std.diff**.
Female sex	56.4	563	58.7	178	61.1	265	0.240	0.12
Arterial hypertension	39.0	391	35.9	109	30.8	135	0.012*	0.11
Diabetes mellitus	12.1	121	10.2	31	10.7	47	0.586	0.05
Chronic kidney disease	3.0	30	4.3	13	4.3	19	0.337	0.10
Coronary heart disease	8.50	85	8.60	26	7.7	34	0.894	0.02
Heart failure	7.7	77	7.9	24	8.4	37	0.882	0.07
COPD	2.9	29	3.0	9	3.9	17	0.593	0.07
In hospital therapy								
Corticosteroids	90.6	908	88.8	270	69.5	306	<0.0001*	0.64
Therapeutic anticoagulation	81.0	812	67.1	204	30.9	136	<0.0001*	1.27
JAK-inhibitors	8.4	84	9.2	28	5.2	23	0.064	0.12
IL6-antagonist	60.9	610	51.3	156	34.1	150	<0.0001*	0.58
Remdesivir	0.3	3	0	0	0.5	2	0.724	0.01
	Median	IQR	Median	IQR	Median	IQR	*P*-value	
Age (years)	59	48–66	56	46–65	55	43–63	<0.0001	0.26
IMPROVE score	1	0–1	0	0–1	0	0–1	0.127	0.17
Creatinine (μmol/l)	89.40	80.60–100.00	91.10	80.90–104.60	90.30	79.95–103.83	0.085	0.08
CRP (mg/l)	26.00	6.00–58.75	26.15	0.00–58.23	18.00	0.00–48.00	0.002	0.32

Baseline characteristics of enrolled patients. COPD, chronic obstructive pulmonary disease; CRP, c-reactive protein; DOAC, direct oral anticoagulation; IL6, interleukine-6; IMPROVE, International Medical Prevention Registry on Venous Thromboembolism; JAK, Janus kinase; std. diff, standardized differences. ^*^p <0.05 using Kruskal-Wallis test or Fisher's exact test.

## Results

**Figure 2:**
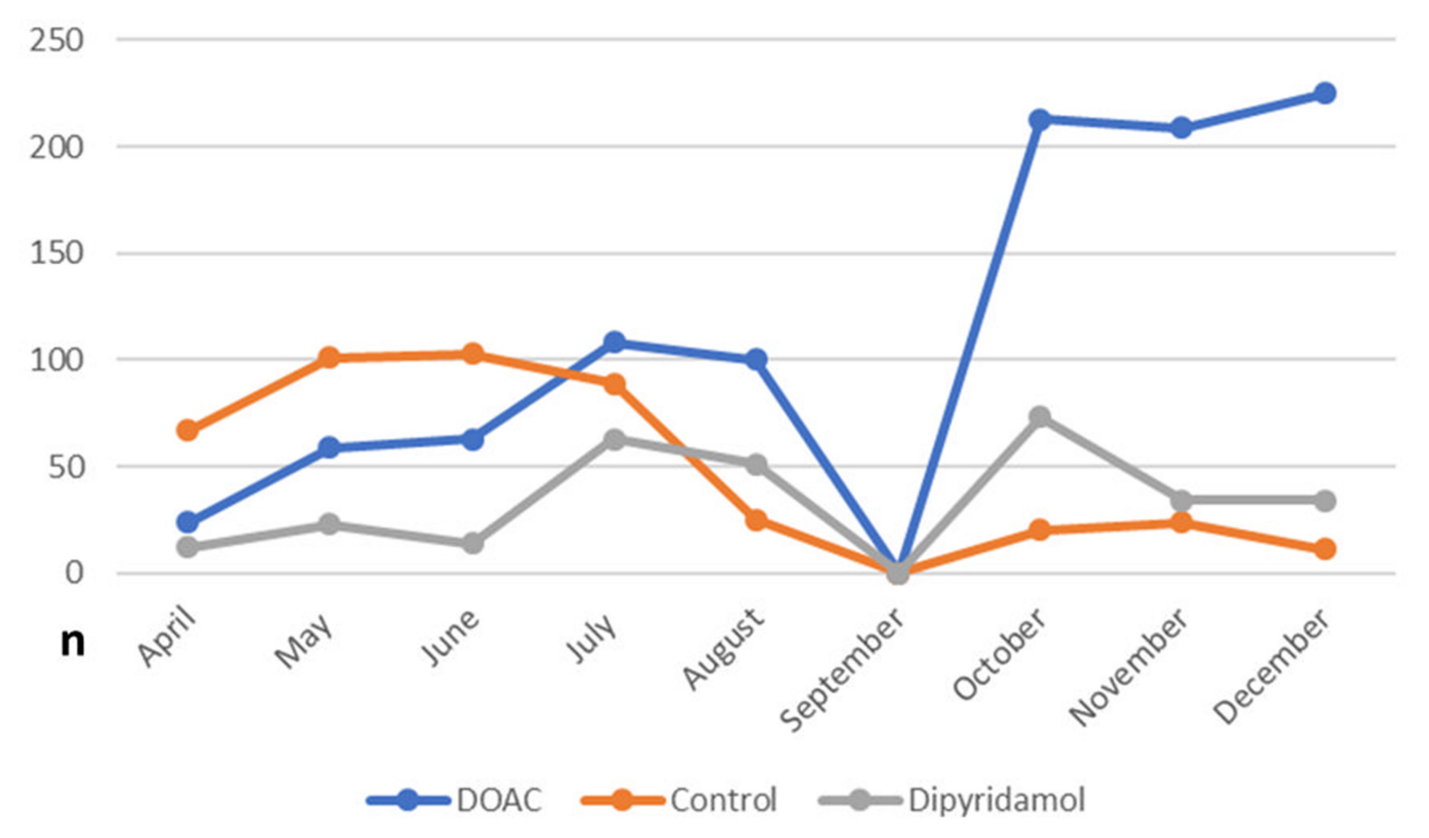
Frequency of prescribed post-discharge antithrombotic regimes in the study population during the study inclusion period (April to December 2020).

In total, 1,746 patients (100% Caucasian) were included in the final statistical analysis. As illustrated in Figure 2, a large number of patients enrolled in the control group were treated in the very early stage of the pandemic, while antithrombotic therapies including DOAC and dipyridamole have been routinely applied since July 2020. Of these, 57.4% (*n* = 1,002) received DOAC (rivaroxaban: 91.6% (918/1,002), 7.1% apixaban (71/1,002) 1.3% dabigatran (13/1,002), and 17.4% (*n* = 304) received dipyridamole. The control group consisted of 25.2% (*n* = 440) of the study population. Baseline characteristics and laboratory values at the time of enrollment are depicted in Table 1. During in-hospital period all patients were treated at least with prophylactic antithrombotic therapy using a heparinoid, the majority also received therapeutic anticoagulation. To note, patients in the DOAC group had a higher prevalence of arterial hypertension and were significantly older than patients in the other groups. Furthermore, patients in the DOAC group significantly more often received corticosteroids, anticoagulation and IL-6 antagonists during the hospital stay (see Table 1).

### Outcome

**Figure 3:**
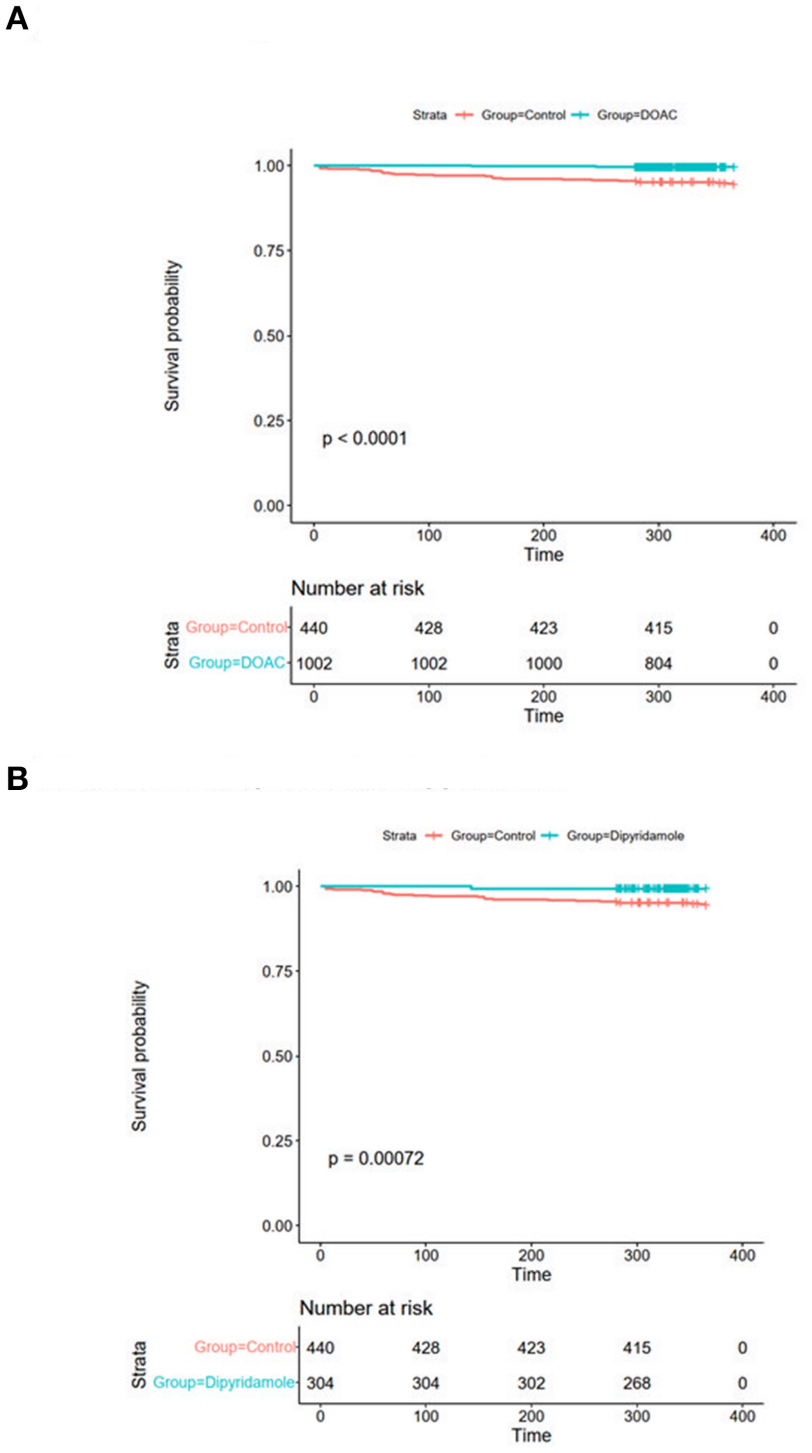
Kaplan-Meier plots of all-cause survival probability **(A)** DOAC: 1.1% vs. Ctrl.: 5.7%, OR 0.19 (95% CI 0.07–0.55), *p* = 0.001 and **(B)** Dipyridamole: 1.2%, Ctrl.: 5.7%, OR 0.20 (95% CI 0.05–0.86), *p* = 0.023.

Mean follow-up in the total cohort was 393 ± 87 days. Patients in the control group had significantly worse 30-day all-cause mortality [DOAC: 0% (*n* = 0), dipyridamole: 0% (*n* = 0), Ctrl.: 0.9% (*n* = 4), *p* = 0.005], 3-month all-cause mortality [DOAC: 0% (*n* = 0), dipyridamole: 0% (*n* = 0), Ctrl.: 2.7% (*n* = 12), *p* < 0.0001], 6-month all-cause mortality [DOAC: 0.1% (*n* = 1), dipyridamole: 0.7% (*n* = 2), Ctrl.: 3.9% (*n* = 17), *p* < 0.0001] and all-cause mortality at the end of follow-up [DOAC: 0.6% (*n* = 6), dipyridamole: 0.7% (*n* = 2), Ctrl.: 5.9% (*n* = 26), *p* < 0.001] than patients treated with DOAC or dipyridamole (see Figure 3; Table 2).

**Table 2:** Outcome of patients enrolled in the three investigated groups.

	**DOAC (*****n*** = **1,002)**		**Dipyridamole (*****n*** = **304)**		**Control (*****n*** = **440)**		
	**%**	***N***	**%**	***N***	**%**	***N***	***P*** **-value**
30-day all-cause mortality	0.0	0	0.0	0	0.9	4	0.005*
3-month all-cause mortality	0.0	0	0.0	0	2.7	12	<0.0001*
6-month all-cause mortality	0.1	1	0.7	2	3.9	17	<0.0001*
Outcomes during total follow-up (393 ±87 days)							
All-cause mortality	0.6	6	0.7	2	5.9	26	<0.0001*
Cardiovascular mortality	0.3	3	0.0	0	2.0	9	0.001*
Myocardial infarction	1.5	15	0.7	2	1.1	5	0.532
Stroke	0.3	3	0.3	1	1.6	7	0.014*
Pulmonary embolism	0.1	1	0.0	0	0.7	3	0.081
Major bleeding	0.0	0	0.0	0	0.2	1	0.426

Outcome of patients enrolled in the three investigated groups. DOAC, direct oral anticoagulation. *p <0.05 using Fisher's exact test.

**Figure 4:**
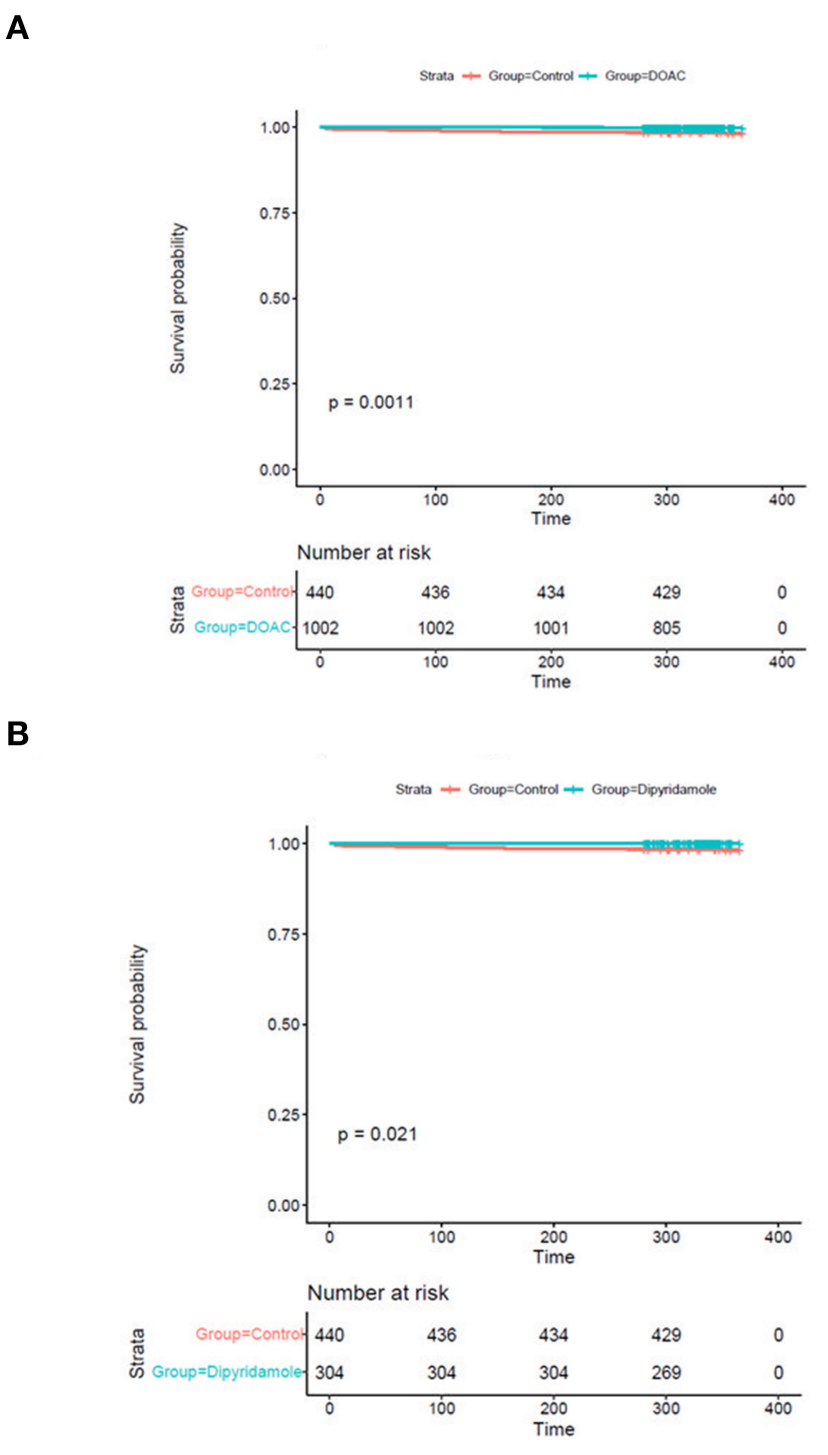
Kaplan-Meier plots of cardiovascular survival probability **(A)** cardiovascular mortality rates of controls vs. patients treated with DOAC, **(B)** cardiovascular mortality rates of controls vs. patients treated with dipyridamole.

**Figure 5:**
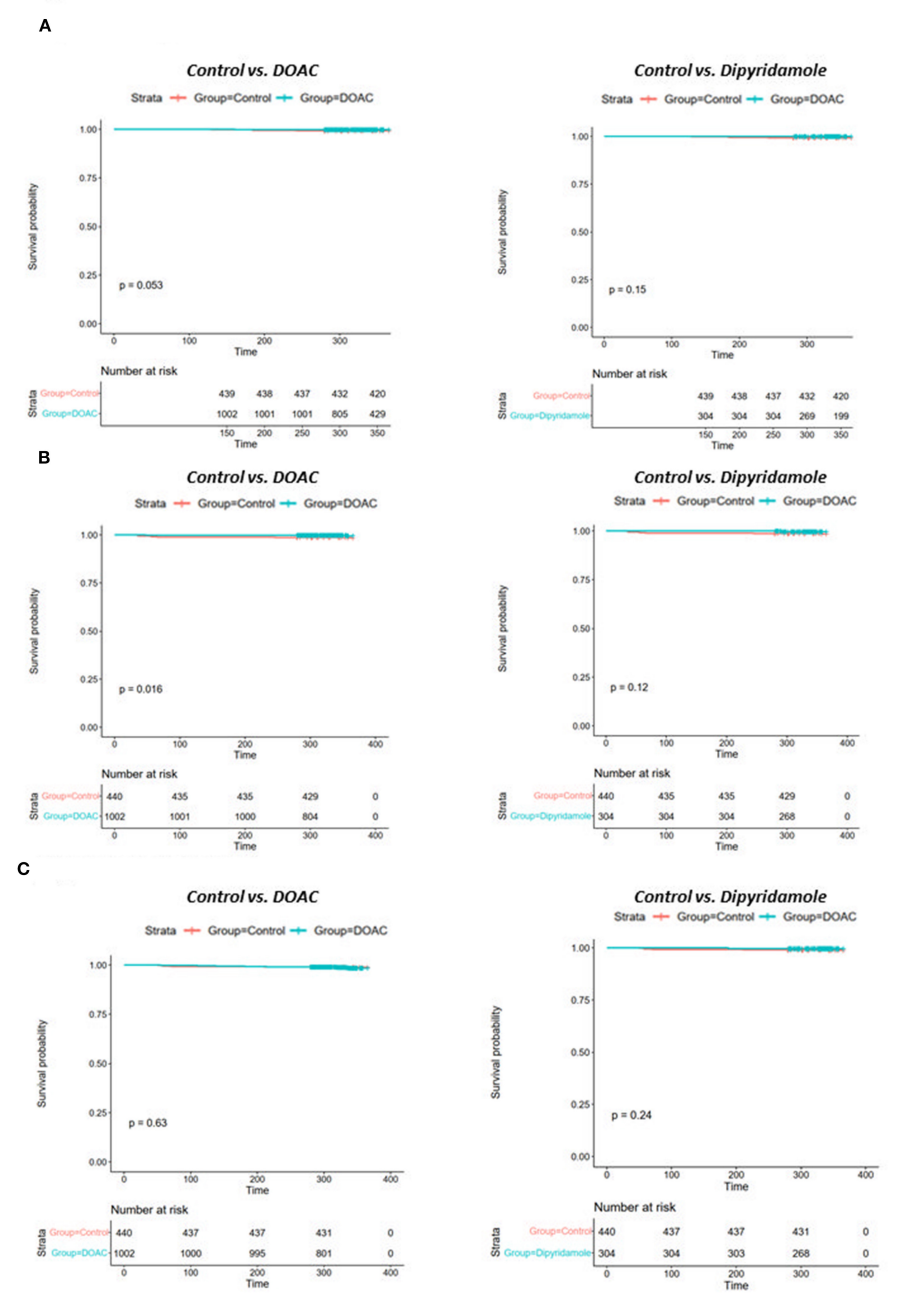
Kaplan-Meier plots of **(A)** pulmonary embolism rates, **(B)** stroke rates and **(C)** myocardial infarction rates of controls vs. patients treated with DOAC and dipyridamole.

While there were no statistically significant differences in the prevalence of myocardial infarction between the three investigated groups, stroke occurred significantly more often in control group patients [DOAC: 0.3% (*n* = 3), dipyridamole: 0.3% (*n* = 1), Ctrl.: 1.6% (*n* = 7), *p* = 0.014] during follow-up. A trend toward higher prevalence of pulmonary embolisms was also observed in the control group [DOAC: 0.1% (*n* = 1), dipyridamole: 0% (*n* = 0), Ctrl: 0.7% (*n* = 3), *p* = 0.081; see **Figure 5**; Table 2], although not statistically significant. Furthermore, cardiovascular mortality was higher in the Crtl: 2.0% (*n* = 9) when compared to DOAC: 0.3% (*n* = 3) and dipyridamole: 0% (*n* = 0, *p* = 0.001, Figure 4; Table 2).

In univariate Cox proportional hazards analysis, both treatment with DOAC or dipyridamole was associated with a reduced risk of mortality [DOAC: HR 0.08 (95% CI 0.03–0.22), *p* < 0.0001; dipyridamole: HR 0.35 (95% CI 0.17–0.72), *p* = 0.005].

**Figure 6:**
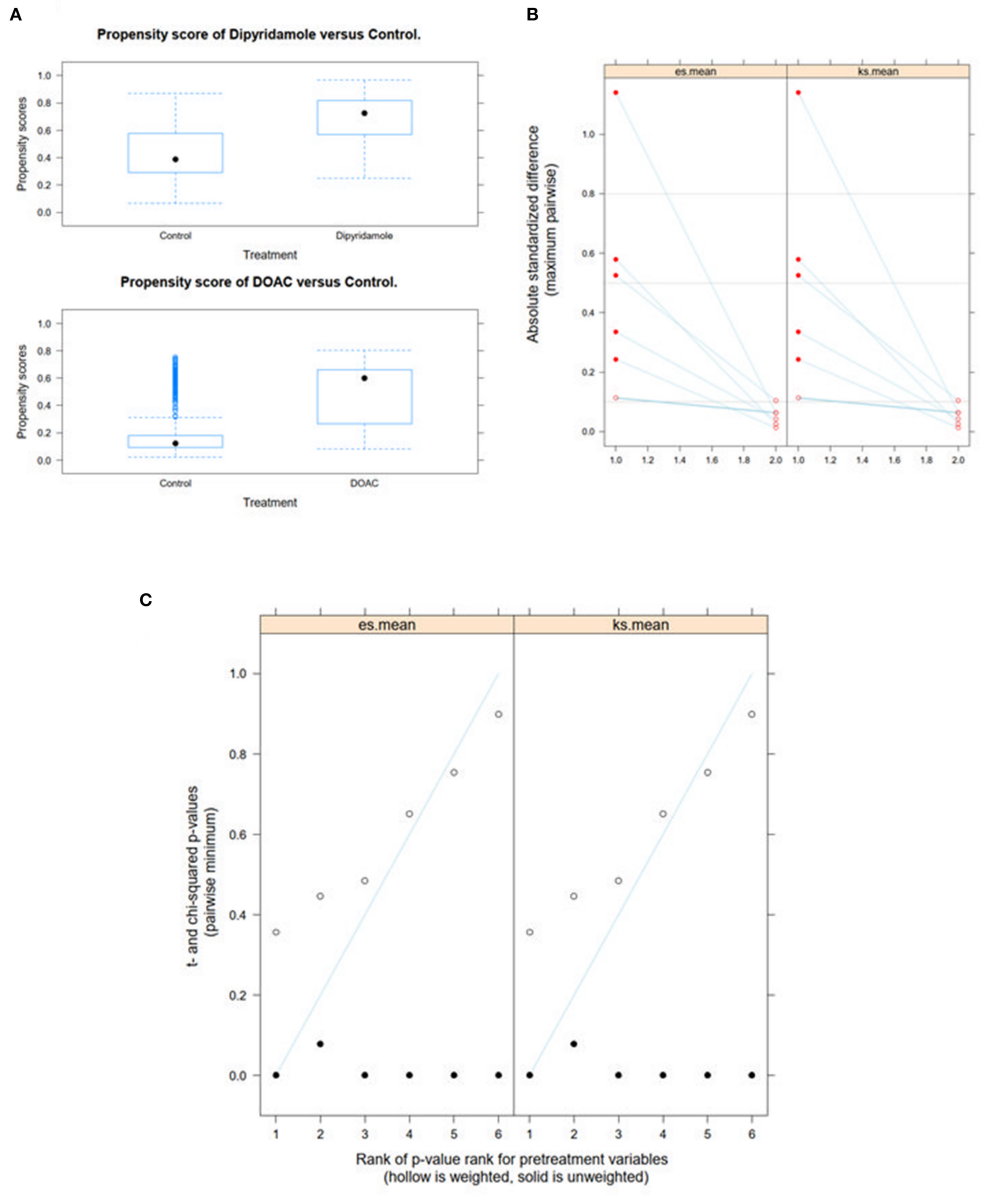
Propensity score weighting of groups was performed by applying Generalized Boosted Models (GBM) using the Average Treatment Effect on Treated (ATT) estimate. **(A)** depicts boxplots of the overlap of propensity score distribution between the three groups, **(B)** the comparison of the absolute standardized mean differences (ASMD) of the selected covariates between the groups before and after weighting and **(C)** the *t*-test and χ2 statistic before and after weighting.

**Figure 7:**
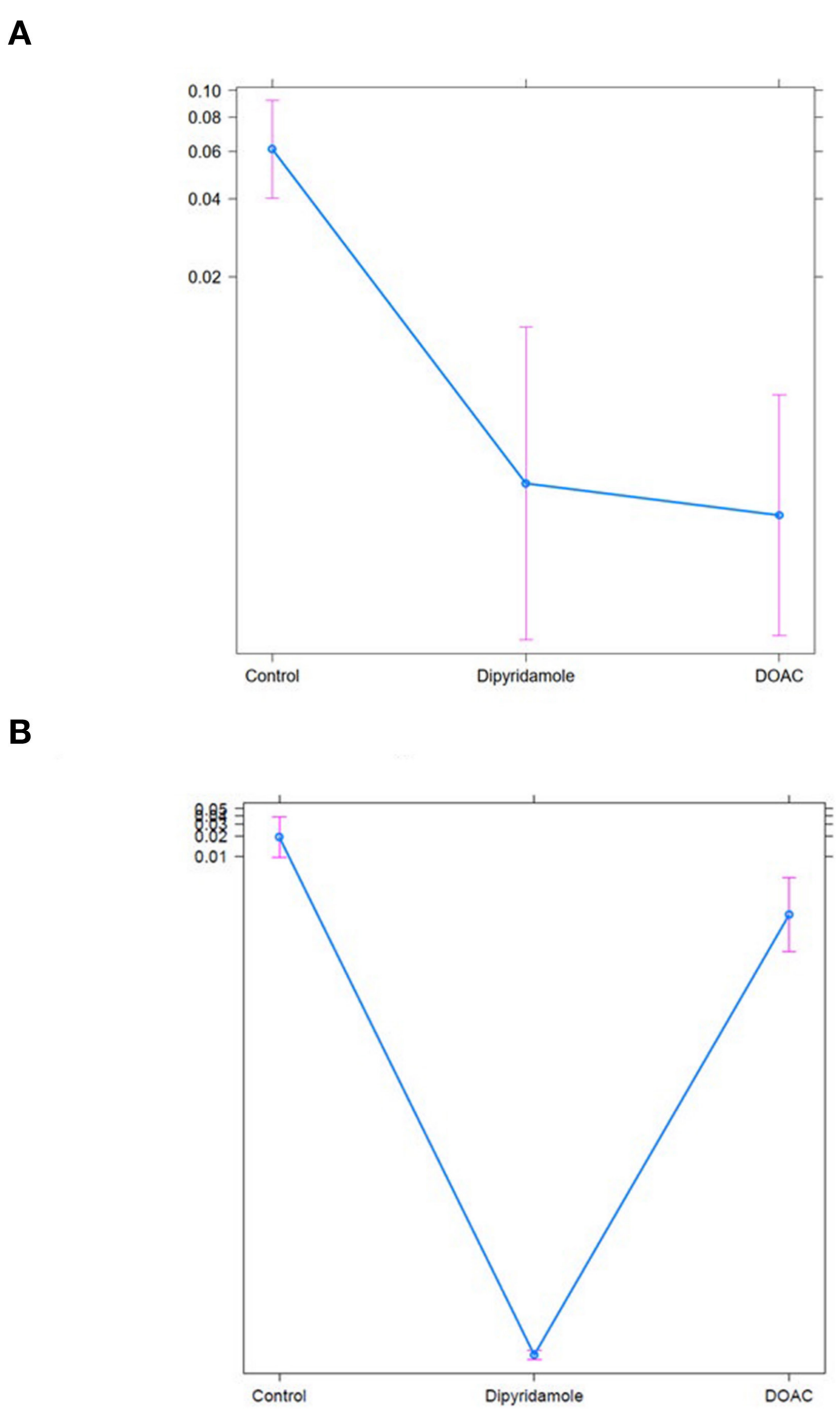
Treat effect plots of weighted binary logistic regression regarding the predefined study endpoints, depicted are predicted probabilities and 95% CI: **(A)** all-cause mortality (predicted probabilities: Ctrl.: 6.1% (95% CI 4.0–9.2) vs. Dipyridamole: 0.3% (95% CI 0.1–1.3) vs. DOAC: 0.2% (95% CI 0.1–0.7), **(B)** all-cause mortality [predicted probabilities: Ctrl.: 6.1% (95% CI 4.0–9.2) vs. Dipyridamole: 0.3% (95% CI 0.1–1.3) vs. DOAC: 0.2% (95% CI 0.1–0.7)], **(B)** cardiovascular mortlaity [predicted probabilities: Ctrl.: 0.02% (95% CI 0.01–0.4) vs. Dipyridamole <0.01% (95% CI 0.0–0.1) vs. DOAC: <0.01% (95% CI 0.0–0.1)].

**Figure 8:**
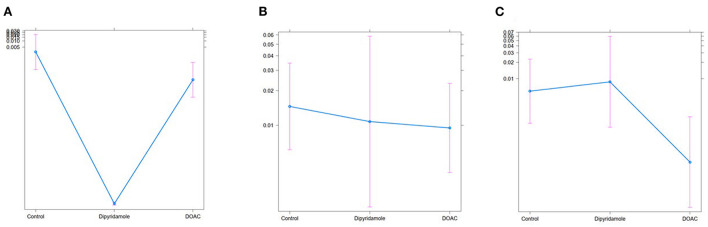
Treat effect plots of weighted binary logistic regression regarding the predefined study endpoints, depicted are predicted probabilities and 95% CI: **(A)** pulmonary embolism [predicted probabilities: Ctrl.: 0.03% (95% CI 0.0–0.2) vs. Dipyridamole: <0.01% (95% CI 0.0–0.1) vs. DOAC: <0.01% (95% CI 0.0–0.1)], **(B)** myocardial infarction [predicted probabilities: Ctrl.: 1.5% (95% CI 0.6–3.5) vs. Dipyridamole: 1.1% (95% CI 0.2–5.9) vs. DOAC: 0.8% (95% CI 0.4–2.3)], **(C)** stroke [predicted probabilities: Ctrl.: 0.6% (95% CI 0.1–2.3) vs. Dipyridamole 0.8% (95% CI 0.1–5.9) vs. DOAC: 0.02% (95% CI 0.0–0.1)].

We performed Generalized Boosted Models (GBM) using the Average Treatment Effect on Treated (ATT) estimate for propensity score weighting of groups to account for covariate imbalances between the three groups, which might affect outcome. Covariates included were those with statistically significant differences and/or standardized differences >0.25 between the groups (see Table 1; arterial hypertension, age, C-reactive protein, in-hospital treatment with corticosteroids, in-hospital treatment with anticoagulation, in-hospital treatment with IL-6 antagonists; see **Figure 6**). After weighted binary logistic regression analysis, the association of treatment with DOAC or dipyridamole and reduced all-cause mortality remained statistically significant [Death during total follow-up: DOAC: B (SE) = −3.33 (0.60), *p* < 0.0001, dipyridamole: B (SE) = −3.04 (0.76), *p* < 0.0001]. In addition, weighted logistic regression revealed protective effects of treatment with DOAC or dipyridamole for cardiovascular mortality [DOAC: B (SE) = −2.69 (0.74), *P* < 0.001, dipyridamole: B (SE) = −17.95 (0.37), *P* < 0.001] as well as for pulmonary embolism [DOAC: B (SE) = −3.12 (1.42), *p* = 0.028, dipyridamole: B (SE) = −17.05 (1.01), *p* < 0.0001]. Treatment with DOAC was furthermore protective for stroke [DOAC: B (SE) = −3.08 (1.23), *p* = 0.0122, dipyridamole: B (SE) = 0.40 (1.23), *p* = 0.743; see also Table 3; **Figures 7, 8**].

**Table 3:** Data of weighted binary logistic regression regarding the predefined study endpoints.

**Dependent variable**	**DOAC**		**Dipyridamole**	
	**B (SE)**	***P*** **-value**	**B (SE)**	***P*** **-value**
Outcome during total follow-up (393 ±87 days)
All-cause mortality	−3.33 (0.60)	<0.0001*	−3.04 (0.76)	<0.0001*
Cardiovascular mortality	−2.69 (0.74)	<0.001*	−17.95 (0.37)	<0.0001*
Myocardial infarction	−0.31 (1.00)	0.757	−0.44 (0.65)	0.498
Stroke	−3.08 (1.23)	0.0122*	0.40 (1.23)	0.743
Pulmonary embolism	−3.12 (1.42)	0.028*	−17.05 (1.01)	<0.0001*

Data of weighted binary logistic regression regarding the predefined study endpoints. DOAC, direct oral anticoagulation; B, regression coefficient; SE, standard error. ^*^p <0.05 using weighted logistic regression analysis.

## Discussion

The post-hospital management of COVID-19 survivors remains a clinical challenge to date. The prothrombotic state, promoted by endothelial inflammation and dysfunction leading to increased platelet adhesion and aggregation as well as proinflammatory cytokine release (1–4) remains a central issue in COVID-19 disease. Meanwhile the rates of thromboembolic events and the use of thromboprophylaxis in hospitalized COVID-19 patients represent a topic of ongoing debate. Although, guidelines on anticoagulation during hospital stay have already been issued, recommendations regarding the antithrombotic treatment for extended post-discharge thromboprophylaxis are conflicting, suggesting either no routine thromboprophylaxis or an individualized approach (23, 24). Of note, existing recommendations focus mainly on anticoagulation, leaving out potential antithrombotic treatment options.

Interestingly, most studies conducted to date reported relatively low rates of thromboembolic events within the first 30–45 days after discharge of hospitalized COVID-19 patients, hence routine thromboprophylactic therapy is not recommended in this patient collective (6, 31, 32). In contrast, the CORE-19 study reported comparably higher rates of thromboembolisms in over three percent of the total patient collective (7). Accordingly, a 46% reduction of major thromboembolic events and death in the presence of (prophylactic) anticoagulation therapy was reported during the mean follow-up of 92 days (7).

Thus, to further investigate efficacy of post-discharge thromboprophylaxis following hospitalization with COVID-19, we analyzed 30-day use of prophylactic DOAC or dipyridamole therapy compared to no anticoagulatory treatment following hospital discharge. To the best of our knowledge, the present study is the first of its kind to offer longer outcome (393 ± 87 days) data capturing extended post-discharge thromboprophylaxis in COVID-19 patients, including different anticoagulatory treatment regimens.

With respect to baseline characteristics, thromboembolic risk as indicated by the IMPROVE score was similar between groups. The control group however showed significantly lower rates of in-hospital corticosteroids, IL-6-inhibitors and therapeutic anticoagulation. This might be due in part to the comparably lower inflammatory burden, indicated by significantly lower baseline CRP-levels in the control group. With regards to concomitant disease, control patients were younger and had lower rates of arterial hypertension (Table 1). However, despite these findings, both DOAC and dipyridamole groups showed lower rates of cardiovascular events during follow-up when matched to untreated patients (Figures 4, 5; Table 2). Importantly, both therapies were associated with reduced all-cause mortality compared to controls, a finding which was consistent during follow-up (30 days, 3 months, 6 months, and overall follow-up; Table 2; Figure 3). Furthermore, cardiovascular mortality was also reduced during follow-up (Figure 4; Table 2). To account for the described differences between groups, propensity score weighting was conducted to account for covariate imbalances, which might affect outcome. As covariates displaying a statistically significant difference were included in the propensity score weighting, the depicted coefficients estimate the causal effects of DOAC or dipyridamole vs. controls assuming there are no unobserved confounders (Figure 6). Of note, the reduction in overall all-cause mortality but also cardiovascular mortality remained highly significant after propensity score weighting of groups (Table 3; Figure 7).

Overall mortality in patients not receiving thromboprophylaxis was high reaching 5.9% during the total follow-up period (Table 2). Thus, mortality rates during follow-up resemble the in-hospital mortality of COVID-19 patients, indicating an ongoing disease process after hospital discharge. This finding could indicate potential severe long-term effects after COVID-19 disease requiring hospitalization. Similarly, the high mortality rates along with the observations of ongoing thromboembolic events during the complete follow-up period might support previously described theories of virus persistence with consequent inflammatory processes suspected in long-COVID disease. Thus, potential beneficial effects of anticoagulatory therapy after hospital-discharge seems plausible. Furthermore, both therapies were also associated with a reduction in several predefined cardiovascular outcomes indicating a link of all-cause mortality to cardiovascular pathologies (Table 3; Figures 7, 8).

While the treatment regime was only applied in the early phase after hospitalization, differences in relevant clinical outcomes were also observed after a longer follow-up. Therefore, it can be speculated that even after survived hospitalization, medical intervention might be crucial to minimize disease progression and improve cardiovascular outcomes as well as mortality rates. Our speculations are further supported by publications indicating an increase in the incidence of cardiovascular events even after mild COVID-19 disease (33) as well as previous reports describing longer virus persistence (34) and hints for inflammatory processes being persistent even during long-term follow-up in COVID-19 survivors (35).

Despite their comparable effects on all-cause mortality and, also cardiovascular mortality, different pathophysiologic effects of DOAC and dipyridamole therapy on predefined cardiovascular events have to be considered with regards to our findings and are potentially in part reflected in our study results.

After propensity score weighting, dipyridamole led to a significant reduction in pulmonary embolism while no significant associations with incidence of stroke and myocardial infarction were evident (Table 3; Figure 8). As dipyridamole acts as an inhibitor of platelet aggregation, a reduction of thrombotic events might be speculated. On the other hand, inflammation constitutes a key player in the pathophysiologic mechanisms leading to thromboembolic events in COVD-19. Sole inhibition of platelet aggregation seems an insufficient explanation on this regard. However, beside the inhibition of platelet aggregation, additional pleiotropic pharmacological actions leading to a broad range of potential beneficial effects in the context of COVID-19 have been reported for dipyridamole, including anti-inflammatory effects along with a significant reduction of D-dimer levels as well as a significant increase in lymphocyte and platelet count (21, 36). Accordingly, the anti-inflammatory effect of dipyridamole might be considered as a potential explanation for the significant reduction of thrombotic and thromboembolic events observed in our study. Additionally, dipyridamole was reported to suppress SARS-CoV-2 replication *in vitro* (21). This is of major importance with respect to the suspected virus persistence in the context of long-COVID-19, with chronically elevated levels of D-dimer and CRP (37). This theory might be supported by the incidence of late thrombotic and thromboembolic events during long-term follow-up after discharge in our study in the control group (Figure 5). Considering these effects, the combination of platelet inhibition, anti-inflammatory effects and a potential impact on virus replication might be speculated to contribute to the observed association between dipyridamole therapy and reduced cardiovascular events observed in post-discharge setting following COVID-19 infection. However, it remains unclear; as towhy no effect of dipyridamole treatment on stroke was observed. A potential explanation is that low-dose dipyridamole monotherapy might have a too small effect on stroke prevention. This is reflected by current recommendations and studies on secondary stroke prevention, in which a higher dose of 200 mg of dipyridamole is recommended only in a combination with acetylsalicylic acid (38). As venous thromboembolisms often occur in the context of COVID-19, potential benefits of dipyridamole therapy is likely decreased in the context of stroke (4).

A significant reduction of stroke and pulmonary embolism rates were observed in patients taking DOAC therapy post-discharge, while no significant association with rate of myocardial infarction was evident (Table 3; Figure 8). Interestingly, studies reported an impact on activation of coagulation in the cytokine storm associated with COVID-19 (14, 39). The thrombin-induced secretion of proinflammatory cytokines and growth factors represent the key factors in coagulation-induced inflammation (40). Consequently, anticoagulation might be helpful to attenuate the interaction between inflammation and thrombosis in COVID-19 (14, 39). However, it can be argued that while anticoagulation is recommended in non-critically ill patients, it failed to provide a clinical benefit in patients requiring intensive care treatment. Nevertheless, a preventive approach must be kept in mind on this regard. While anticoagulation might attenuate the vicious circle of thrombosis and inflammation, the process might be too far advanced in severe COVID-19, requiring intensive care treatment. Accordingly, the potential anti-inflammatory effect of anticoagulation therapy might be negligible in the context of advanced cytokine storm and high inflammatory burden. This may explain the failure of previous multicentre studies on therapeutic anticoagulation in intensive care COVID-19 patients. Of note, patients in the present study received prophylactic DOAC doses to counterbalance thromboembolic and bleeding risk.

While no significant differences in major bleeding were observed in the two treatment arms, one major bleeding was observed in the control group during follow-up (0.2%, *P* = 0.426; Table 2). However, it must be mentioned that minor bleeding events could not be assessed given the study design. Thus, the validity of our study findings with respect to the bleeding endpoint is limited.

In summary, the present study is the first to offer long follow-up (393 ± 87 days) of different thromboprophylactic treatment regimens after hospitalization for COVID-19. Mortality rates were significantly reduced by both 30-day regimes of dipyridamole and prophylactic DOAC treatment, emphasizing the ongoing thromboembolic and inflammatory burden in COVID-19 in the early post-discharge period following the acute phase of the disease. Accordingly, thromboprophylactic treatment might offer beneficial effects in the long-term treatment of COVID-19 patients. Therefore, further randomized trials are necessary to investigate the effects of these regimes in COVID-19 survivors.

## Limitations

The present study has by design its limitations, mainly due to its single-center and retrospective design as well as lack of randomization and treatment arm blinding. Among others, this could bias the results due to hospital-specific standards of patient care. The overstrained medical system amidst the pandemic may have exacerbated cardiovascular events rates and mortality leading to an overestimation of the effects of the investigated medical regimes. On the other hand, rates of cardiovascular outcomes were based on hospitalized events only. Therefore, an underestimation of events is possible. This may be further aggravated by the unwillingness of patients to be hospitalized during the pandemic. While anticoagulatory regimes in the investigated center were used as the pandemic progressed, a large number of patients enrolled in the control group were treated in the very early stage. Therefore, limited accumulated clinical experience, the implementation of novel therapy regimes and the evolution of the viral genome could have affected disease management and therefore long-term outcomes. Nevertheless, to adjust for this bias, propensity score weighting of groups was performed, which did not significantly affect our results. Furthermore, since the first novel viral variants, B.1.1.7 and B.1.351 were declared a variant of concern on December 18th, 2020, followed by P.1 on January 11th, 2021 (41) differences in the viral genome seem improbable in our study cohort which was recruited between middle of April 2020 and December 2020. Based on our study design, we were only able to analyze bleeding events requiring hospitalization, which is a major limitation of our study. Nevertheless, the low incidence of bleeding events observed in our trial, seems plausible, since it is comparable to results presented in the MICHELLE study, which applied a similar therapeutic regime in a comparable patient population (26). Moreover, it is important to emphasize that our findings only apply to patients hospitalized with moderate COVID-19 infection.

## Data availability statement

The raw data supporting the conclusions of this article will be made available by the authors, without undue reservation.

## Ethics statement

The studies involving human participants were reviewed and approved by Bashkir State Medical University (N5, 2020). Written informed consent for participation was not required for this study in accordance with the national legislation and the institutional requirements.

## Author contributions

All authors listed have made a substantial, direct, and intellectual contribution to the work and approved it for publication.

## Funding

This study was supported by grant of Russian Science Foundation No. 22-18-20123.

## Conflict of Interest

The authors declare that the research was conducted in the absence of any commercial or financial relationships that could be construed as a potential conflict of interest.

## Publisher's note

All claims expressed in this article are solely those of the authors and do not necessarily represent those of their affiliated organizations, or those of the publisher, the editors and the reviewers. Any product that may be evaluated in this article, or claim that may be made by its manufacturer, is not guaranteed or endorsed by the publisher.
