# Supplementary figures and images for: Early antithrombotic post-discharge therapy using prophylactic DOAC or dipyridamole improves long-term survival and cardiovascular outcomes in hospitalized COVID-19 survivors
Source: Front Cardiovasc Med. 2022 Jul 29;9:916156. doi: 10.3389/fcvm.2022.916156 (PMC9372296; doi:10.3389/fcvm.2022.916156)

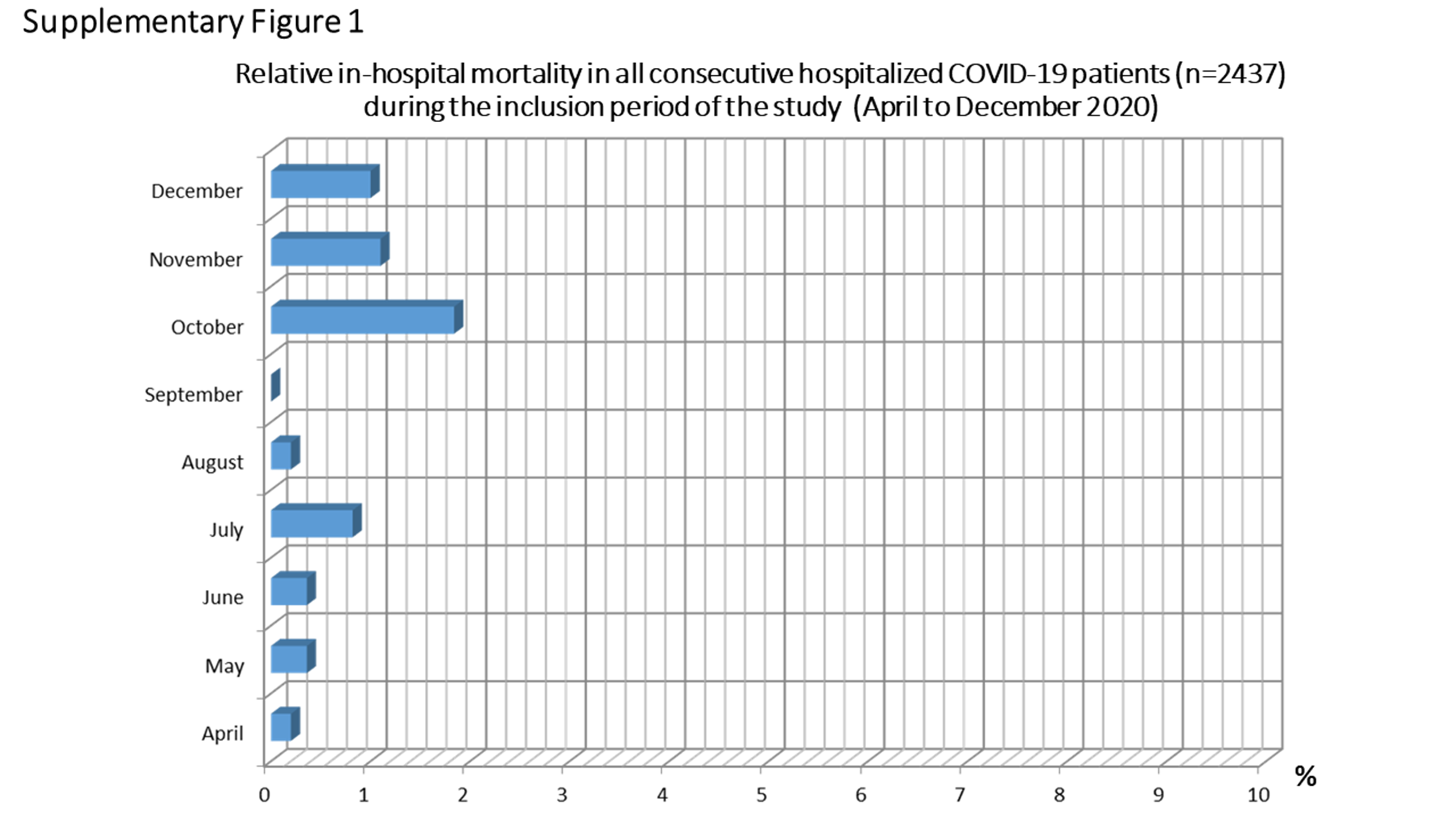

Supplement: Supplementary file 1 [file Image_1.TIF]
